# Exercise Interventions for the Treatment of Affective Disorders – Research to Practice

**DOI:** 10.3389/fpsyt.2014.00046

**Published:** 2014-05-06

**Authors:** Robert Stanton, Brenda Happell, Melanie Hayman, Peter Reaburn

**Affiliations:** ^1^Institute for Health and Social Science Research, Centre for Mental Health Nursing Innovation, School of Nursing and Midwifery, Central Queensland University, Rockhampton, QLD, Australia; ^2^School of Medical and Applied Sciences, Central Queensland University, Rockhampton, QLD, Australia

**Keywords:** exercise, mood disorders, physical activity, treatment, mental health

## Introduction

Mental illness presents a growing disease burden, with worldwide prevalence estimates between 18 and 36% (1). In the USA, the prevalence of affective disorders including unipolar depression and bipolar disorder (BD) is around 20% (2, 3). While psychotropic medications remain at the front line of treatment for affective disorders, a growing body of research evidence strongly supports the role of exercise in the treatment of these affective disorders. Although remaining to be elucidated, there are a number of potential mechanisms by which exercise may be beneficial including neurobiological (4, 5) and pharmacological-like mechanisms (6). In the present paper, we shall discuss recent findings from systematic reviews and make recommendations for structured exercise, as distinct from unstructured or incidental physical activity, in the treatment of both depression and BD. This review also examines the role of exercise in the treatment of post-natal depression (PND) since this often transient but prevalent condition is rarely examined.

## Exercise for Depression

Three recent systematic reviews (7–9) confirm the potential benefit of exercise for people with depression. Rethorst et al. (7) reviewed 75 RCTs comparing exercise versus no treatment or wait-list control. Fifty-eight of the 75 studies were included in a meta-analysis, which showed a clinically significant effect of exercise on depressive symptoms (ES: −0.80, 95% CI −0.90 to −0.67). More recently, Rimer et al. (8) reported that both aerobic exercise and muscle strengthening exercises are effective in reducing depressive symptoms with resistance exercise (ES: −0.85, 95% CI −1.85 to −0.15) or combined aerobic and resistance exercise programs (ES: −0.82, 95% CI −1.39 to −0.25) showing larger effect sizes, albeit with wide confidence intervals. In further support of the role of aerobic exercise in the treatment of depressive symptoms, Robertson et al. (9) also reported a significant, large effect (ES: −0.86, 95% CI −1.12 to −0.61) of walking on symptoms of depression.

In contrast, not all reviews support the role of exercise in the treatment of depression. Krogh et al. (10) reported only limited benefit [Standardized Mean Difference (SMD): −0.40, 95% CI −0.66 to −0.14)] for exercise in a population diagnosed with clinical depression. When only studies with long-term follow-up data were analyzed, this effect disappeared (SMD: −0.01, 95% CI −0.28 to 0.26) suggesting no long-term benefits of exercise for people with depression. More recently, Cooney et al. (11) concluded that while exercise is more effective in the treatment of depression compared to no treatment (SMD: −0.62, 95% CI −0.81 to −0.42), it is no more effective than antidepressant medications (SMD: −0.11, 95% CI −0.34 to 0.12) or psychological treatment (SMD: −0.03, 95% CI −0.32 to 0.26). When the analysis was limited to high quality studies with long-term follow-up, the positive effects of exercise were markedly diminished.

Despite recent systematic reviews and meta-analyses producing contrasting outcomes, it would appear that exercise may be as effective as other treatment strategies and better than no intervention at all. Importantly, people with mental illness, including those with depression, view exercise as a valuable yet underutilized treatment strategy, and this acceptance of exercise treatment may lead to better long-term adherence (12).

As far as we are aware, only three reviews have examined the exercise program variables (frequency, intensity, duration, and type of exercise) for the treatment of depression (7, 13, 14). These are highlighted in Table 1. The most recent of these reviews examined exercise program variables from studies reporting positive mental health outcomes for people with depression. Stanton and Reaburn (14) reported that supervised aerobic exercise, performed in either group or individual formats, undertaken 3–4 days per week, at low to moderate or self-selected intensity, for 30–40 min per session for at least 9 weeks, is likely to provide positive benefits in the treatment of depression. This is not substantially different to the exercise recommendations for healthy populations (15). Therefore, clinicians should be confident that public health exercise guidelines represent an excellent starting point when prescribing exercise for people with depression.

**Table 1:** **Summary of exercise prescription guidelines**.

Author	Frequency (per week)	Intensity	Session duration	Mode of exercise
**DEPRESSION**				
Rethorst et al. (7)	3	Not reported	45–60 min	Not reported
Perraton et al. (13)	3	60–80% HR_max_	30 min	Individualized according to preference
Stanton and Reaburn (14)	3–4	Low-moderate or patient preferred	30–40 min	Any aerobic activity
**HEALTHY POPULATIONS**				
Garber et al. (15)	≥5	Moderate	Min 30 min/session or ≥150 min/week	Individualized according to preference
	≥3	Vigorous	Min 20 min/session or ≥75 min/week	
			Or a combination to achieve ≥500–1000 kCal/week	
**POST-NATAL DEPRESSION**				
Lewis and Kennedy (16)	3	Moderate	40 min	Pram-walking
**BIPOLAR DISORDERS**				
Alsuwaidan and McIntyre (29)	5–7	Moderate	30 min	Not reported

## Exercise for Post-Natal Depression

Two recent reviews offer support for exercise in the treatment of PND (16, 17). Lewis et al. (16) reviewed nine studies, including four 12-week RCTs investigating the effect of exercise on PND. Three of the four RCTs reported an improvement in PND symptoms (18–20) and one reported no effect of exercise (21).

Most recently, Blamey et al. (17) reported a moderate (SMD: 0·73, 95% CI 0.35–1.11) but not clinically significant (<4 points on Edinburgh PND Scale) effect of exercise in the treatment of PND. The authors also reported that structured exercise classes were more effective than tailored advice. However, the high risk of bias, poor reporting of data, and substantial study heterogeneity limits the interpretation of these findings.

Not all reviews support the effectiveness of exercise in the treatment of PND. Daley et al. (22) reviewed five RCTs comparing exercise of any type, with any other treatment, or no treatment and reported no significant effect of exercise as a stand-alone intervention, compared to no exercise in women with PND (SMD: −0.42, 95% CI −0.90 to 0.05). However, based on the limited available evidence, exercise appears to be beneficial in the treatment of PND (23) and has been included in clinical practice guidelines for the treatment of PND (24). However, this recommendation is based on two small, low quality trials (18, 19) and does not offer detailed guidance on exercise prescription. Possibly due to the lack of high quality RCTs and the significant heterogeneity in intervention design, no systematic reviews have examined the exercise program variables for the treatment of PND. From the review of Lewis et al. (16), a 12-week program of pram-walking including social support may be valuable for the treatment of PND as outlined in Table 1. Until more high quality RCTs are conducted to examine the appropriate program variables for exercise in the treatment of PND, the recommendations of Stanton and Reaburn (14), or the exercise program variables used for healthy populations, might be considered valuable.

## Exercise for Bipolar Disorder

Compared to other affective disorders such as depression, there is considerably less research examining the effect of exercise in the treatment of BD. However, from the findings of studies undertaken to date, it would appear that exercise may reduce both depression and anxiety (25), improve acute wellbeing (26), and reduce stress (25, 27) in people with BD. As with other studies of exercise in the treatment of mental illness, the limited number of studies on exercise intervention and BD are typically underpowered, have poor adherence and poor reporting of program variables. This makes replication and interpretation of study outcomes difficult.

To our knowledge, only one systematic review has been conducted on the effect of physical activity and exercise in people with BD (28). From the six studies included, the authors concluded that, while exercise may be feasible, more research is necessary before recommendations can be made for appropriate exercise prescription in individuals with BD. In a review of the neurobiological correlates to BD, Alsuwaidan and McIntyre (29) concluded that a program of exercise consistent with the public health dose of 30 min of moderate intensity exercise on most days of the week may be effective for individuals with BD (see Table 1). This recommendation was based on the findings from both animal and human studies, which show exercise may mediate the neurobiological dysfunction seen in people with BD.

Despite the research limitations, people with BD report that exercise assists with managing symptoms, except during periods of mania or severe depression (30). Experiences from people with BD suggest a need for flexibility to tailor exercise to the mood state (e.g., more rhythmic exercise during periods of mania) since exercise may exacerbate manic symptoms or result in injury due to over-exertion (30). A more recent study (31) has confirmed these views, showing lower levels of exercise were associated with higher levels of depression, while higher levels of exercise were associated with more symptoms of mania. Supervised individualized exercise delivered by experienced exercise specialists may minimize the risk of adverse events during these times.

## Limitations Associated with Interpretation of the Literature

A number of systematic reviews and meta-analyses have highlighted significant methodological weaknesses in many of the previously published studies including a lack of blinding to treatment, lack of blinding of assessors, poor reporting of program variables, inconsistent use of assessment tools and high dropout (10, 32). More specifically, questions arise regarding the lack of comparison groups (7), lack of long-term data (10), significant heterogeneity (8, 16), and low sample sizes (9, 16). These limitations should be considered when interpreting the outcomes of systematic reviews and meta-analyses. Importantly, future studies should ensure these limitations are addressed in the design, implementation, and analysis of exercise interventions for people with affective disorders.

## Research to Practice

At present, the findings from systematic reviews reporting the effect of exercise on depression, PND and BD are somewhat conflicting. However, from the available evidence, exercise prescription based on current public health recommendations (15), the reviews of Lewis et al. (16), and Alsuwaidan and McIntyre (29), or the recommendations for people with depression (14), are likely to offer positive benefits with few adverse events.

In summary, supervised, individual, or group cardiovascular exercise, performed at low to moderate or self-selected intensity, for 30–40 min per session, with three to four sessions per week, over at least 9–12 weeks, is likely to be beneficial for people with affective disorders. The mode of cardiovascular exercise should be individualized according preference and access to resources. Walking or cycle exercise, pram-walking or other supervised group or individual cardiovascular exercise, is likely to be effective. The unique states of hypo- and hyper-mania should be considered when prescribing exercise for people with BD. It is our conclusion and recommendation that for effective exercise prescription, interdisciplinary care teams, comprising accredited exercise physiologists, physical therapists, mental health nurses, case managers, psychologists, and psychiatrists should strive to implement these guidelines to improve the mental and physical health of this vulnerable population.

## Conflict of Interest Statement

The authors declare that the research was conducted in the absence of any commercial or financial relationships that could be construed as a potential conflict of interest.
